# At the crossroads of infection and malignancy: the challenge of tuberculosis in migrating populations – Case Report and Epidemiologic Analysis

**DOI:** 10.1186/s12879-025-12043-6

**Published:** 2025-12-23

**Authors:** Hans-Jörg Epple, Teresa Domaszewska, Ann-Christin von Brünneck, Christian Furth, Steffen Dommerich, Christoph Lange, Dinah von Schöning, Georg Maschmeyer, Lena Bös, Stefan Schwartz, Thomas Schneider, Stephan Mathas

**Affiliations:** 1 Department of Gastroenterology, Rheumatology and Infectious Diseases, Charité – Universitätsmedizin Berlin, Corporate Member of Freie Universität Berlin and Humboldt-Universität zu Berlin, Berlin, Germany; 2 Respiratory Infections Unit, Department for Infectious Disease Epidemiology, Robert Koch Institute, Berlin, Germany; 3 Institute of Pathology, Charité - Universitätsmedizin, Berlin, Corporate Member of Freie Universität Berlin and Humboldt-Universität zu Berlin, Berlin, Germany; 4 Department of Nuclear Medicine, Charité – Universitätsmedizin, Berlin, Corporate Member of Freie Universität Berlin and Humboldt-Universität zu Berlin, Berlin, Germany; 5 Department of Otorhinolaryngology, Charité – Universitätsmedizin, Berlin, Corporate Member of Freie Universität Berlin and Humboldt-Universität zu Berlin, Berlin, Germany; 6 Division of Clinical Infectious Diseases, Research Center Borstel, Borstel, Germany; 7 German Center for Infection Research (DZIF), Partner Site Hamburg-Lübeck-Borstel-Riems, Borstel, Germany; 8 Respiratory Medicine & International Health, University of Lübeck, Lübeck, Germany; 9 Baylor College of Medicine (BCM), Houston, TX USA; 10 Department of Microbiology, Labor Berlin-Charité Vivantes GmbH, Berlin, Germany; 11 Department of Hematology, Oncology and Cancer Immunology, Charité – Universitätsmedizin Berlin, Corporate Member of Freie Universität and Humboldt-Universität zu Berlin, Berlin, Germany; 12 Max-Delbrück-Center for Molecular Medicine in the Helmholtz Association (MDC), Biology of Malignant Lymphomas, Berlin, Germany; 13 Experimental and Clinical Research Center (ECRC), A joint Cooperation Between Charité and MDC, Berlin, Germany; 14 German Cancer Consortium (DKTK), Partner Site Berlin, A Partnership Between DKFZ and Charité – Universitätsmedizin Berlin, Berlin, Germany

**Keywords:** Tuberculosis, Case report, Malignancy, Migration, Langerhans

## Abstract

**Introduction:**

*Mycobacterium tuberculosis* infection might result in fatal outcome in patients with haematologic malignancies or those with primary or iatrogenic immunodefects. However, in countries with low tuberculosis (TB) incidence the awareness for TB is still challenging, with an increasing need due to migrating populations from regions with high TB to low TB incidence countries.

**Case report and epidemiological analysis:**

A 52-year-old caucasian female, refugee from Afghanistan, presented at University Hospital Charité (Berlin, Germany) with progressive left-sided hearing loss due to ear canal obstruction as well as right axillary exophytic and ulcerating skin lesions, finally diagnosed as *BRAF*-V600E-mutated Langerhans cell histiocytosis (LCH). Following systemic chemotherapy, LCH showed complete remission (CR) with however unexpectedly progressive left-axillary lymphadenopathy. Biopsy of these lymph nodes revealed granulomatous inflammation with central caseous necroses. *Mycobacterium tuberculosis* DNA was undetectable in a biopsy from the lesion, but culture of fresh re-biopsy material showed growth of *M. tuberculosis*. Given the patient´s migration history from a high-prevalence (Afghanistan) to a low-prevalence (Germany) TB country, we also present the long-time trend of the total number of TB patients notified in Germany in 2002–2023, i.e. a total of 21 years, aggregated by country of birth. Whereas the number of TB patients born in Germany is decreasing since 2002, the number of patients born abroad exceeded the number of those born in Germany in 2012, and remained higher ever since.

**Conclusion:**

Our report highlights the challenge to treat patients at the crossroads of malignancy and TB, and the need for appropriate attention and awareness of physicians of the increased TB risk in people migrating from a high- to a low-burden TB country. Also, the case demonstrates once more the high value of culture for diagnosis of mycobacteria infection.

**Clinical Trial:**

Not applicable.

## Introduction

Patients with haematologic malignancies or primary or iatrogenic immunodefects are prone to mycobacterial infection including tuberculosis (TB) caused by *Mycobacterium tuberculosis*, with putative fatal outcome [1]. In countries with low TB incidence (defined as < 10 TB cases/100,000 population) [2] - such as most countries of the European Union and European Economic Area - clinical awareness for TB but also its diagnostics are still challenging, with an increasing need due to populations migrating from regions with high TB incidence and/or affected by crises [3]. Here, we report on the management of a refugee treated at University Hospital Charité (Berlin, Germany) for Langerhans cell histiocytosis (LCH), a rare haematologic malignancy considered to be derived from myeloid precursor cells, who experienced reactivation of TB under chemotherapy. To draw attention to rising numbers of patients in corresponding situations, we present epidemiological data on foreign-born TB patients in Germany that were notified within national TB surveillance over the past 21 years according to their country of birth. With this report, we aim to sharpen the awareness of haematooncologists for the increased number of patients with TB with a migration history from high- to low-TB-burden countries [4, 5], and thus for their increased risk of TB reactivation during immunosuppressive therapy. This report also highlights that, consistent with Koch´s postulates [6], even in times of advanced molecular diagnostics culture remains an essential, highly sensitive, and specific method for the detection of mycobacteria.

## Case report and epidemiological analysis

**Fig. 1:**
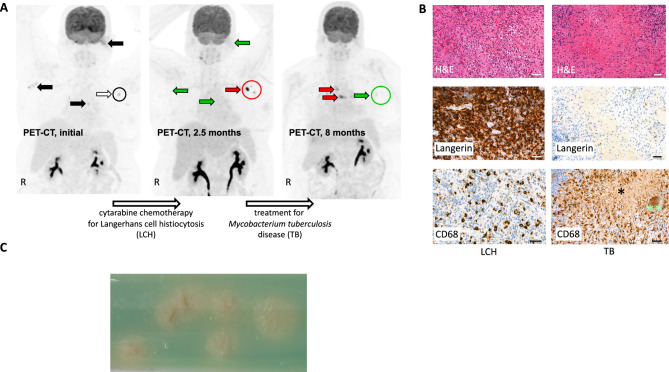
PET-CT scan showing course and pathology and microbiology findings of a patient with LCH and TB. (**A**) PET-CT at diagnosis (left; 03/2023), after 3 cycles of cytarabine chemotherapy (center; 05/2023), and after TB treatment for 4 months (right; 8 months after initial PET-CT, 11/2023). Initial PET^+^ lesions are marked by black and white arrows (left). After chemotherapy, LCH lesions in metabolic remission are marked by green arrows; the progressive left axillary lymph nodes are marked by a red arrow and circle. R, right. The progressive left axillary lymph nodes were punctured for further diagnostics. (**B**) Conventional and immunohistochemistry stainings of LCH (left column; left mastoid sample) and TB (right column; left axillary lymph node). Top row, hematoxylin and eosin (H&E) staining; center and bottom, stainings for Langerin and CD68, respectively. Note, that LCH shows mononuclear cells with eosinophilic cytoplasm (H&E) and presence of eosinophilic granulocytes, strong reactivity for Langerin (center) and partial positivity for CD68 (bottom; infiltrating macrophages are strongly CD68^+^), whereas the left axillary lymph node biopsy shows the typical features of TB with granulomatous inflammation with central caseous necrosis (*), epitheloid cells and Langhans giant cells (**) forming granulomas. Scale bars, 50 µm. (**C**) Subculture of *M. tuberculosis* isolate on Löwenstein-Jensen medium showing the typical aspect of rough, nonpigmented colonies (with permission by Inna Friesen, National Reference Center for Mycobacteria, Research Center Borstel, Germany)

A 52-year-old caucasian female, refugee from Afghanistan, presented in February 2023 at Charité with progressive left-sided hearing loss due to ear canal obstruction and mastoid cell-destruction as well as right axillary exophytic and ulcerating skin lesions. F18-fluorodeoxyglucose (FDG)-positron emission tomography-computed tomography (PET-CT) showed FDG-avid lesions - indicating increased metabolism - at these sites (left mastoid, standard uptake value (SUV) max. 12; skin lesions, SUV max. 9), and also in mediastinal (SUV max. 7) and left axillary lymph nodes (SUV max. 10) (Fig. 1A, left). The patient underwent left-sided mastoidectomy, and in this sample as well as from right axillary skin biopsy, a diagnosis of *BRAF*-V600E-mutated LCH was established (Fig. 1B, left). According to sites and organs affected, the disease was classified as multifocal and multisystem LCH. Based on clinical presentation and international treatment recommendations [7], systemic chemotherapy with cytarabine (100 mg/m^2^ IV, d1-5, every 4 weeks) was initiated. After 3 cycles, hearing fully recovered and right-axillary skin lesions showed complete remission (CR). PET-CT-scan confirmed CR in all lesions apart from deep left axillary lymph nodes, in which unexpectedly progressive lymphadenopathy and increased metabolic activity (SUV max. 15,5) were noted (Fig. 1A, center).

Histopathology of a formalin-fixed paraffin-embedded (FFPE) ultrasound-guided left-axillary lymph node biopsy revealed granulomatous inflammation with central caseous necroses, Langhans giant cells and epitheloid granuloma (Fig. 1B, right). However, Ziehl-Neelsen staining did not show acid-fast bacilli, and PCR-based analyses (according to Schewe et al. [8] followed by subtyping using VisionArray MYCO 2.0 (Chipron)) did not detect *M. tuberculosis*-specific DNA. In addition, also retrospectively the patient was not aware of previous contacts to persons with confirmed TB infection or screening investigations specific for TB. Also, neither a chest X-ray performed approximately 6 months before diagnosis of LCH nor baseline imagings (PET-CT-scan/CT) for LCH did show any findings (such as apical scarring, calcified leasons or pleural thickening) suggestive for old, inactive TB. Therefore, screening – including interferon-gamma release assays (IGRA) - for latent TB infection was not performed before initiating chemotherapy for LCH.

Yet, as the pathology report was not consistent with LCH but highly suspicious for a granulomatous disease, e.g. TB, re-biopsy of the axillary lymph nodes was performed. This time, not only microscopy using Auramine staining (Kreienbaum Neoscience) and PCR for detection of *M. tuberculosis* complex DNA in fresh biopsy material (cobas MTB test, cobas 8800 system, Roche), but also mycobacterial culture on both liquid and solid media (BACTEC MGIT 960 culture system, Becton Dickinson, and Stonebrink agar and Löwenstein-Jensen agar, Artelt-Eclit) were performed. While acid-fast-bacilli were still not visible by microscopy and *M. tuberculosis*-specific DNA remained to be undetectable, liquid culture showed growth of *M. tuberculosis* after 14 days of incubation (Fig. 1C, subculture on solid media). Genotypic susceptibility testing using the line probe assay GenoType MTBDRplus (Hain Lifescience) did not detect mutations associated with resistance to rifampicin (R) or isoniazid (H). Liquid drug susceptibility testing for H, R, pyrazinamide (Z) and ethambutol (E) was performed using the automated BACTEC MGIT 960 system (Beckton Dickinson) with full drug susceptibility of the isolate.

Accordingly, and in line with the current WHO recommendations [9], the patient was started on a body weight adapted 6-month HRZE treatment regimen, and treatment for LCH was interrupted to avoid further immunosuppression. After 10 days of therapy, the patient developed muscle and joint pain of the upper and lower extremities without elevation of inflammatory markers and a moderate increase of serum uric acid to 9.6 (normal range < 6.1) mg/dl. In addition, the patient had developed a moderate eyelid eczema. Self-medication with non-steroidal anti-rheumatic (NSAR) drugs had not sufficiently controlled the muscle and joint symptoms but was associated with abdominal discomfort. Intolerance to Z-medication was assumed, and therefore Z was terminated, and the tuberculosis therapy was transiently continued as R, H, E. After discontinuation of Z, serum uric acid returned to normal values, and the symptoms of the patient including the eyelid eczema completely resolved. Based on an individual decision by the managing physician guided by extrapolating guideline recommendations for therapy of R-susceptible/H-resistant tuberculosis [10–12], Moxifloxacin (MOX) was added. The 4-drug regimen (H, R, E, MOX) was continued for 2 months followed by 4 months of R and H without any further significant adverse effects. PET-CT-scan at the end of treatment showed full regression of the TB-affected lymph nodes (Fig. 1A, right), and the patient had recovered from fatigue. A right-sided subtle, partially nodular thickened, clinically inapparent basal pleural effusion was considered to be most likely a paradoxical reaction due to the anti-tuberculosis therapy.

However, PET-CT-scan also showed new mediastinal FDG-avid lesions (Figs. 1A, right). Histologically, mediastinal relapse of LCH was proven, without any evidence for TB. We initiated LCH-treatment with methotrexate (MTX; 20 mg/m^2^ weekly). We favored this treatment regimen - which is primarily used for skin-limited LCH [13] - due to its putatively less pronounced immunosuppressive effect given the previous history of TB reactivation. During the first 3 months of MTX treatment, anti-tuberculosis therapy with H and R was continued. PET-CT-scan in june 2025 showed complete remission of the previously known LCH lesions, and the patient presented in good condition.

**Fig. 2:**
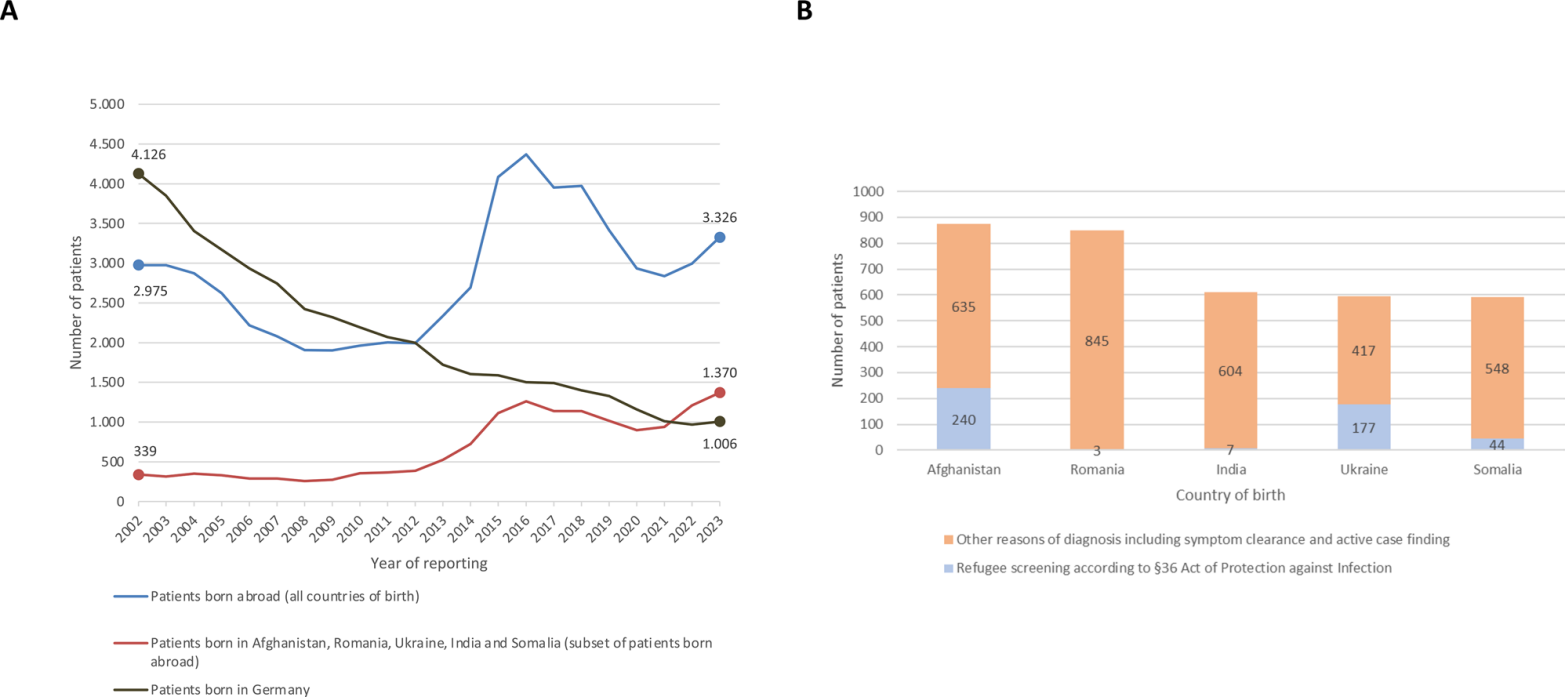
Epidemiological data of TB patients notified to the RKI within national surveillance in Germany 2002–2023. (**A**) Total numbers of TB patients, stratified by country of birth in 2002–2023. Curves represent patients born in Germany (grey curve) vs patients born abroad (blue curve) vs patients from the 5 most frequently reported foreign countries of birth of all TB cases notified in Germany 2021–2023 (orange curve; including Afghanistan). Note, that the number of TB patients born in Germany is decreasing since 2002, while the number of patients born abroad exceeded the number of patients born in Germany in 2012 and remained higher ever since. x-axis, years of reporting; y-axis, number of patients. (**B**) Total number of TB patients by case-finding (refugee screening according to §36 of the German Act of Protection against infection [obligatory screening upon admission to community centers, e.g. accommodation centers], marked in blue, vs other reasons of case findings, marked in orange) for the 5 most frequently reported foreign countries of birth of all TB cases notified in Germany in 2021–2023. x-axis, country of birth; y-axis number of patients

Given the patient´s migration history from a high-prevalence TB country (Afghanistan, TB incidence of 180/100,000 population in 2023 according to WHO; ref. [14]) to a low-prevalence TB country (Germany, TB incidence of 5.3/100,000 population in 2023; ref. [15]), we present the long time trend of the total number of TB patients in Germany that were notified to the Robert Koch Institute (RKI) in 2002–2023, i.e. a total of 21 years, in the context of national TB surveillance and according to the German Protection against Infection law (Infektionsschutzgesetz), and analyzed it with the special focus on case finding [16]. The results were aggregated by country of birth in the following manner: patients born in Germany *versus* abroad *versus* born in the 5 most frequently reported foreign countries of birth - including Afghanistan - of all TB cases notified in Germany in 2021 to 2023 (Fig. 2A). Whereas the number of TB patients born in Germany has been decreasing since 2002, the number of patients born abroad exceeded the number of those born in Germany in 2012, and remained higher ever since. In line with increased immigration to Germany from crisis-affected regions, the number of foreign-born patients peaked in 2015–16 (in particular due to the instable political situations in countries such as Syria and Afghanistan) followed by a transient decrease until 2020–21, and has been on the rise again since 2022 (mainly due to the Ukraine war). As depicted in Fig. 2B, only a small proportion of TB patients was diagnosed following screening upon admission of refugees or asylum seekers to community accommodation centers according to §36 of the German Act of Protection against Infection (https://www.gesetze-im-internet.de/ifsg/__36.html).

## Discussion

###  TB reactivation under immunosuppression 

Our report highlights the difficulties to identify and manage patients at the crossroads of malignancy and TB, two potentially fatal diseases [1]. LCH lesions are composed of LCH cells surrounded by various immune cells, and elevated levels of inflammatory cytokines are detectable in the serum of LCH patients, but there are no obvious data on general immune defects in LCH patients [17, 18]. It is thus more likely that the chemotherapy for LCH impaired proper immune cell function and likely promoted TB reactivation, a situation which required multidisciplinarity in diagnostics and treatment encompassing haematooncologists, infectious disease specialists, radiologists and microbiologists. Our case illustrates how the missed TB detection at the initiation of immunosuppressive treatment resulted in TB reactivation, which prevented full dose treatment continuation for LCH. Although subsequent treatment with MTX resulted in complete remission of LCH in our case, such treatment interruptions might result in disease progression by more aggressive malignancies. Prior screening and preventive treatment for latent tuberculosis might have prevented active infection requiring subsequent antiinfective combination therapy and should be considered in high-risk patients with haematological malignancies, but the available data do not strongly support this approach [1].

### Limitations of molecular diagnostics

The automated cobas MTB PCR assay used in this study for TB detection showed recently high sensitivity results in respiratory samples, comparable to other nucleic acid amplification tests such as Xpert MTB/RIF [19]. However, particularly for extrapulmonal, paucibacillary tuberculosis as in our patient case, molecular diagnostic approaches might fail to detect TB, and thus culture should always be performed in parallel [20, 21].

### Immigrant populations as TB high-risk group

The presented epidemiological data from Germany covering the past 21 years highlight the fact that appropriate attention and awareness of physicians and other healthcare providers of the increased TB risk in people migrating from a high- to a low-burden TB country is essential to detect TB cases early, even years after migration and despite lacking evidence of TB during the initial screening. There is substantial evidence that people migrating from a high-prevalence TB country to the European Union, European Economic Area or the UK have a higher risk for active tuberculosis and worse TB treatment outcomes than the respective host population [3]. However, the benefit of general screening approaches for patients with haematologic malignancies is not consistantly evident [1], but should be considered at low-threshold – including IGRA - in patients migrating from high-risk TB countries. Obviously, a screening approach based solely on chest X-ray fails to identify individuals with latent or extrapulmonary TB. If some of them, like our patient, eventually reactivate TB, only high-level of awareness ensures timely diagnosis and treatment of affected patients and prevents fatal outcomes as well as secondary spread of TB infection.

In Germany, §36 of the German Protection against infection law (https://www.gesetze-im-internet.de.ifsg/__36.html) mandates TB screening for migrants being admitted to shared accommodation facilities. This typically involves a chest X-ray, particularly for adults, and aims to identify infectious TB cases. Other than that, the same screening recommendations apply to migrants as to the general population (screening for TB infection with IGRA test is recommended e.g. for people with suspected TB exposure as part of contact investigation, with planned immunosuppressive anti-TNF treatment, or people with first HIV diagnosis) [22]. National guidelines for TB screening in migrants are currently being developed [23]. However, even best evidence-based recommendations will not cover every specific patient and disease-related circumstances, which is why the doctor´s awareness of TB, careful consideration of patient-specific risk factors (such as origin, migration route, malnutrition, co-morbidities, planned intervention) and considering TB as differential diagnosis is important. The limitations of our study include data completeness of the German national surveillance data, as the case finding variable illustrated in Fig. 2 was notified for less than 90% of the patients over the years 2002–2023. Since obligatory screening in Germany applies only to refugees and asylum seekers admitted to community accommodation centers, the number of patients diagnosed *via* screening does not allow to draw conclusions regarding screening efficacy.

Together, our report highlights the need for the awareness of TB in patients migrating from high-prevalence to low-prevalence TB countries. Especially in case of specific interventions which can lead to TB progression, TB screening should be considered carefully for the migrant patients with higher TB risk, e.g. originating from high-incidence countries or refugees with possible TB exposure during migration routes. The diagnostic results in this case demonstrate once more that, even in times of advanced molecular diagnostics, culture still remains a solid and reliable diagnostic standard for detection of mycobacteria.

## Data Availability

Patient case: Not applicable. Epidemiological data: Aggregated notification data can be requested from the authors or the Research Data Management Unit of the Robert Koch Institute (MFI@rki.de) under reasonable conditions.
